# Capitalizing on Synergies—A Discourse Analysis of the Process of Collaboration Among Providers of Integrative Health Care

**DOI:** 10.1371/journal.pone.0122125

**Published:** 2015-03-20

**Authors:** Susanne Andermo, Tobias Sundberg, Christina Forsberg, Torkel Falkenberg

**Affiliations:** 1 Karolinska Institutet, Department of Neurobiology, Care Sciences and Society, Division of Nursing, 141 83, Huddinge, Sweden; 2 I C—The Integrative Care Science Centre, 153 91, Järna, Sweden; Canadian Agency for Drugs and Technologies in Health, CANADA

## Abstract

**Background:**

Integrative health care (IHC) combines therapies and providers from complementary and conventional health care. Previous studies on IHC have shown power relations between providers but few studies have explored how the interaction develops over time. The objective of this study was to explore the development of IHC collaboration and interaction among participating providers during a series of consensus case conferences for managing patients with back and neck pain.

**Methods:**

This qualitative study was conducted within a pragmatic randomized controlled clinical trial in primary care. Patients' treatment plans were developed based on IHC provider consensus conferences (n = 26) of which 15 (5 of the first, 5 in the middle, and 5 of the last in the clinical trial) were selected for analysis. Findings were derived by means of discourse analysis, focusing on the participants’ use of subject positions during the conferences.

**Findings:**

The IHC team in this study gradually formed a group identity, moving their subject positions from individual treating subjects to members of a team and were able to make consensus-based decisions about patients’ individual treatment plans. In the discourse, the IHC team identified collaborative shortcomings and problematized the provision of IHC. They were able to capitalize on the synergies in their collaboration and developed a shared vision of IHC provision.

**Conclusions:**

The process of IHC collaboration involved the gradual formation of an IHC team identity, which facilitated interdisciplinary, non-hierarchical consensus-based decision-making in the team. The discourse further suggests that a reform of some legal and organizational health sector barriers might be needed to realize sustainable implementation of IHC services in Sweden.

## Introduction

The World Health Organization (WHO) recognizes that many health care systems are fragmented; they emphasize a need for collaborative education and practice to strengthen health systems and improve health outcomes [1]. Mickan et al [2] show that team work in interdisciplinary health care can improve both patient satisfaction and health outcomes. Integrative health care (IHC) aims to adhere to the same high standards of service as those provided by conventional care but at the same time integrating selected complementary therapies. Zollman and Vickers [3] define complementary and alternative medicine (CAM) as *“Complementary and alternative medicine (CAM) is a broad domain of healing resources that encompasses all health systems*, *modalities*, *and practices and their accompanying theories and beliefs*, *other than those intrinsic to the politically dominant health system of a particular society or culture in a given society”*. The objective in IHC is to provide patient-centred, personalised, safe and effective health care with as few side-effects as possible [4]. In recent authoritative recommendations, such as the Beijing declaration [5] and World Health Assembly resolutions [6], the WHO urges member states to integrate evidence- based complementary medicine into their national health systems, i.e. IHC. Correspondingly, IHC services are increasingly becoming part of health care policy and research agendas [7–9], and recommendations for IHC strategies can be found in national care guidelines relating to mental and physical disorders such as depression and chronic pain, whereby mindfulness, massage and manual therapy may be included in the care for certain diagnoses [10, 11]. Collaboration within IHC is a complex field, with many different theoretical descriptions of clinical provision [12]. Boon et al. [12] identify seven conceptual models of integrative health care: parallel, consultative, collaborative, coordinated, multidisciplinary, interdisciplinary, and integrative. The conceptual models represent a position on a continuum ranging from non- integrative to fully integrative.

Despite the mushrooming of IHC services across the world, from numerous Chinese Traditional Medicine hospitals in China and Anthroposophic Medicine hospitals in Europe to most large university hospitals in the USA that offer IHC services [13] and a growing number of initiatives in Africa, research analyzing the process of IHC collaboration and interaction is generally lacking. The few research reports that do exist show evidence of power struggles between complementary providers and conventional providers, leading to the adoption of a predominantly biomedical language to steer such collaborations [14–16]. For example, Shuval et al [14] describe how complementary providers are not accepted as real staff members in Israeli hospitals. Hollenberg [15] addresses the process of interaction between biomedical and complementary providers in two Canadian clinics. His findings reveal patterns of biomedical dominance in the collaboration. Anderson [16] demonstrates how alternative or non-Western theories are expressed, but not discussed or contested; instead, a biomedical language is used in a test panel with IHC providers. Kaboru [17] shows a similar problem among traditional and biomedical health workers in Zambia, where collaboration is described more as one-way bio medically oriented teaching rather than two-way learning. Gaboury [18], however, points out that providers within integrative health care in Canada do not view communication as a major problem for collaboration. D´amour [19] suggests two key elements in successful collaborative practices, viz. to address and serve the complexity of clients’ needs and to serve professional needs by integrating each team member’s perspectives in the construction of a respectful and trusting team. From a patient perspective, Frenkel et al [20] emphasizes that it is in the patient´s interest that the complementary- and conventional providers are able to work as a team, with an open dialogue and a high degree of professionalism between them.

There is a shortage of research analyzing the process of IHC collaboration and interaction. An understanding of how an IHC model develops, when it comes to the actual process of interaction and collaboration, can contribute to the exploration of outcomes of IHC interventions and facilitate the development of future integrative care collaborations in line with WHO recommendations. The IHC model examined in the present study was developed as a patient-centered interdisciplinary, non- hierarchical collaboration of conventional and complementary therapies [21]. The model has a similar structure as the interdisciplinary and integrative model developed by Boon et al [12] and emphasizes that the team members respect each other and that each provider can contribute to the care with their particular knowledge [12]. The objective of the study was to explore how the interaction and collaboration developed between IHC providers participating in a series of case conferences for consensus-based management of patients with back and neck pain during a pragmatic randomized controlled clinical trial in primary care.

## Methods

### Study design

The investigation used a qualitative study design based on a social constructivist worldview, i.e. where a person’s experience as a member of a society or a group shape their view of the world [22]. The study deals with the complex field of IHC collaboration, where each provider represents different professional forms of care, e.g. western, Chinese and Japanese medical therapies, as well as social categories such as class and gender. Furthermore, medical knowledge and practice vary in time and place, being part of a specific historical and social context [23, 24].

In the communication between the IHC providers, the discursive practice is interpreted as a social and dynamic process that creates meaning in different situations [25]. Discourse is defined as “a textual unit larger than a sentence” [26]. Subject positions are used in discourses and defined as “`locations within conversation” [27]. The significance of subject positions is not fixed; instead, a person uses multiple and temporary fixations of subject positions in different discourses to capture the meaning of their identity [28, 29]. Wodac [30] discusses how the discourse construction of *us and them* is a base for forming identity. The “othering” is also described as an antagonism that helps to stabilize the discursive system [28].

### Study setting

The study was conducted alongside a pragmatic randomized controlled clinical trial, registration number NCT00565942, which evaluated the comparative effectiveness of the IHC intervention compared to standard primary care [21, 31, 32]. The IHC team consisted of one general provider and 8 senior CT providers: three Swedish massage therapists, a manual therapist/ naprapath, two shiatsu therapists, an acupuncturist and a qigong therapist. All providers except one were native Swedish people who worked with complementary therapies that have their origins in different cultures. There were equal numbers of female and male CT providers and they were aged between 30 and 50 at the beginning of the study. The CT providers were recruited based on their experience within their respective CT field and their experience of sharing cases with conventional providers [21]. All had many years’ experience in their respective field and some had dual training. The conventional provider who participated in the patient conferences had an interest in complementary medicine and had previous training in e.g. kinesiology, although he now only practiced conventional care. The health care providers were invited to participate in a clinical research project on the development, implementation, and evaluation of an integrative medicine model for managing patients with back and neck pain in Swedish primary care.

In the clinical trial, patients were randomized to receive either conventional care (n: 36) or IHC (n: 44). The patients were males and females between 18 and 65 years of age who were experiencing non-specific back/neck pain of sub-acute to chronic duration. Patients with severe causes of back and neck pain, including specific pathologies such as fractures, infections, disc hernias, cancer, or osteoporosis, were not included. Twenty-six patient conferences were held and equally distributed over a period of 1 year and 4 months. At the patient conferences, the IHC team discussed treatment strategies for the patients. The patient conferences were scheduled for two hours and lasted between 30 minutes and 2 hours. Typically, at least 6 of 8 providers participated in each patient conference. Most of the conferences were conducted in a separate conference room at one of the participating primary care units. On one occasion, a conference was conducted at the research department at the university and on another occasion at one of the providers’ private clinic.

Participating patients were included continuously during the whole project and received on average seven CT treatment sessions over a period of 10 weeks during the clinical trial [31].The patients did not participate in the patient conferences due to logistical and clinical constraints; instead it was considered that they could take part in the health care process through personal interaction with the providers on the IHC team.

### Data Collection

Data was collected at all patient conferences. No question or discussion guide was used; natural occurring conversations between the IHC providers were instead digitally recorded and collected as data for the study. When selecting data material for analysis, the inclusion criteria were based on the ability to observe the development of the IHC team’s discussions by selectively choosing different time periods for the conferences. Fifteen conferences were selected for analysis, divided into the first five, five in the middle, and the last five conferences. The reason for choosing five conferences at each time was to be able to follow the discourse about newly included patients as well as to follow the discourse about some of the patients’ whole intervention period.

### Data analysis

The data were analyzed using discourse analysis, drawing on discursive psychology [25, 27] and discourse theory [28, 29, 33]. The patient conferences chosen were transcribed verbatim and spot-checked for accuracy. When the transcripts were finalized, the identities of all patients and providers were removed. The providers’ names were changed to CAM provider 1–8. The transcripts were analyzed in their original language. The transcripts were first read several times to get a sense of the whole as well as differences between the first, the middle, and the last patient conferences. The analysis began with a broad pragmatic coding focusing on the interaction and collaborative process between the IHC providers. The coding was made as inclusive as possible [25], coding both the process of collaboration and how the providers described and talked about their collaboration. Analytical questions arose during the coding process and guided the analysis. The first question was, “How is the process of decision-making structured and how does it develop?” The analysis focused on the collaborative patterns in the decision-making process by looking at how the providers made treatment suggestions, whose suggestions were chosen, and how the providers responded and received response from the other team members. Patterns were sought in the data, looking at both differences and consistency in the collaborative patterns between the different conference occasions. As patterns emerged, more analytical questions were used to guide the analysis: How do the therapists represent themselves? How are the therapists’ individual roles negotiated in the IHC collaboration? How do the therapists see the role of the IHC team in a larger medical discourse? The concept of subject positions [25, 27–29] was used to see how the individual providers positioned themselves, each other, and the IHC team. The patterns that emerged are described as themes and sub-themes in the result section.

### Ethics

The patients in the study were informed about the study by their general physician; if they were interested the research co-coordinator gave them verbal information about the study and sent out information to those interested in participating. The patients returned signed consent forms. The participating providers were given verbal and written information about the study. Verbal informed consent from the providers was electronically documented due to the busy clinical environment. The research project was approved by the regional ethics committee at Karolinska Institutet (Dnr: 668–03, 650–04, 121–32).

## Results

The overarching result is described in the theme “The process of collaboration” with three sub- themes: “Building consensus-based decision-making”, “Building a team” and “The role of integrative health care”. Sub-themes describe the collaborative process at the different IHC team conferences (Table 1).

**Table 1:** Themes and sub- themes.

Patient conferences:	In the beginning (1–5):	In the middle (6–10):	In the last (11–15):
Theme1: The process of collaboration			
Sub- theme 1.1: Building consensus-based decision-making	A slight biomedical dominance in decision-making	Consensus-based integrative decision-making	
Sub- theme 1.2: Building a team	Positioning themselves as individual provider subjects. Emphasizing individual therapies and roles. Learning from each other’s methods	Positioning themselves as members of a team—Capitalizing on the knowledge and strength in the IHC collaboration	
		Confirming and contesting each other’s informed discussions. Finding their different roles	
Sub- theme 1.3: The evolving role of integrative health care	Searching for a goal for IHC	Emphasizing the importance of complementary therapies	Defining and discussing the goal of IHC
		Positioning the IHC team as an enchanted choice to conventional health care	
	Discussing collaborative shortcomings		

### Sub-theme 1.1: Building consensus-based decision-making

The theme “Building consensus-based decision making” describes how the providers position themselves in the decision-making process and how they construct a non-hierarchical form of decision-making. The process of decision-making involved the physician introducing and medically describing the patient for the IHC team, followed by an interdisciplinary discussion and a joint decision on a recommended treatment for the patient. At the first patient conferences, most endorsed decisions were based on the physicians’ initial treatment suggestions. The physician used the pronoun “I” in the discourse:

Excerpt 1.

*Physician—“I am willing to give the patient to either one of you”*.

By using the pronoun “I” in the sentence, the physician positioned himself as a leader in the decision-making process. After the initial treatment plan decision, one IHC provider usually began treating the patient. At the next conference, the provider responsible for each patient presented the current treatment and the outcome was evaluated by the IHC team. The patient could then continue with the provider, change to another or receive treatment from two or more providers at the same time. At the first patient conferences, some providers were more involved in the discussion than others. For example, one of the CAM providers gave many treatment suggestions at the first patient conferences, but his/her suggestions were often not commented on by the other team members.

At the middle and last patient conferences, the IHC team providers’ involvement increased and they made more suggestions:

Excerpt 2:

CAM provider 1—I think acupuncture …

Physician- Should we take acupuncture first? ….

Project leader- If CAM provider 3, if you start, how quickly should we follow up with structural assessment?

CAM provider 2- It is possible at the same time.

CAM provider 3- It can be done in that way.

In excerpt 2, the discourse was dynamic: CAM provider 1 gave a suggestion that the physician accept by forwarding the question to the team. The physician is not then taking a leading role. The project leader follows up with a question about a structural assessment (lines 3–4). Both of the CAM providers answer that it is possible without the need for further explanation of the meaning of a structural assessment. At the middle and last patient conferences, the providers both contested and agreed with each other’s treatment suggestions and the IHC team made consensus-based decisions concerning the patients’ treatment plans.

### Sub-theme 1.2: Building a team

The theme “Building a team” describes a process of change in positioning where the IHC providers first position themselves as individual provider subjects and how they later, in the middle and last conferences, change their position to that of members of a team. Their position as individual provider subjects at the first conferences is shown when they explain and emphasize their individual therapies to each other. Discussions about the content of each provider’s therapeutic activities are common. In excerpt 3, one of the CAM providers explains how his/her dual competence influences the practice:

Excerpt 3:

CAM provider 3- No, I am a …therapist as well.

CAM provider 2- and she has to forget about that now.

CAM provider 3- So, I think about energy as well.

CAM provider 1- But does it matter?

CAM provider 3- I am a … therapist here, but I have to take in a little of this, I think.

Excerpt 3 shows the CAM provider 3 is seeking recognition for her dual competence. When CAM provider 2 says that she has to forget about her dual competence, the provider is expressing an assumption that they have to stay within a certain role in the IHC team. CAM provider 3 continues to seek recognition for an energy perspective (line 3) and explains that her dual practice would influence her practice (line 5) even when CAM provider 1 asks if it matters. In this discourse, the IHC provider is negotiating her individual role in the collaboration.

The providers begin to notice the benefits of the collaboration:

Excerpt 4.

CAM provider 5- I could learn from the questions (in the journals) and CAM provider 1 can be useful to me, I can learn a lot and that feels incredible fun.

Project leader- Yes, thanks for that. It is important to bring these issues with journal documentation up and how one can communicate.

*CAM provider 3- When CAM providers 7 and I went through what she had written; she was also a bit worried*. *Will they understand what I have written*? *Some things they might not understand*, *I said*, *and some things will be easier to understand*. *We will see*, *it is also something to learn from*.

Physician- Because I believe we have done a bit of the development in this group.

CAM provider 5—I have worked so long, and no one has even cared about how I write, I only write (journals) for myself.

Excerpt 4 shows a discourse about sharing written documentation where the IHC begins to value the collaboration, observing that they can learn from each other. CAM provider 5 begins by emphasizing the process of learning as something useful and fun. The project leader confirms that it is important to bring this aspect up. CAM provider 3 expresses both a concern whether the others will understand the written information and a possibility to learn from each other by referring to a conversation with another CAM provider. The physician gives assurance to the process by emphasizing the development within the team. CAM provider 5 describes previous experiences where no one had “even cared” that are described as lonely.

At the middle patient conferences, the IHC providers begin to position themselves as members of a team. They have learned more about each other’s methods and are able to have more informed discussions about the patients. A part of the team-building process is when the providers begin to understand and explain how their different roles relate to each other.

Excerpt 5

CAM provider 1- I see a role that has started to happen here… I see the manual therapy provider relying on anatomy and physiology, kind of a link between conventional and complementary health care.

Excerpt 5 shows how one of the providers was perceived as having a role in between conventional health care and complementary health care. The team-building process is further shown in the discourse as the providers have moved from an emphasis on their own part in the IHC treatment to the importance of the IHC collaboration:

Excerpt 6

CAM provider 4- One feels wow, thanks for this project, think if CAM provider 6 had been standing in the room next to, every day. We could have done like this all the time. How many like this have I finished after four times and it did not get better and now it became something different and I can meet that result. I have no need any more to fix it by myself, I only want her to get better, so it was really good.

In excerpt 6, CAM provider 4 is emphasizing the importance of the collaboration in the IHC team by saying that he has no need to fix it by himself. By comparison with previous experience, the ability to collaborate provides strength and solutions for the patients. At the last patient conferences, the providers continue to position themselves as members of a team:

Excerpt 7

CAM provider 5- She had booked an appointment with a physical therapist and I thought, open, it is really good. Try that to I said. When we were done, she had understood what this group can do, that there are people who know this. She started to become doubtful about if she was going to go to the physical therapist. I encouraged her, I said go there and say the same things and we will see.

Excerpt 7 shows how CAM provider 5 both encourages the patient to go to a physical therapist and at the same time emphasizes the strength of the IHC team. This strength is implicitly described through the patient being described as having doubts about going to someone outside the team after she had understood what the IHC team can do.

### Sub-theme 1.3: The evolving role of integrative health care

The sub-theme “The evolving role of integrative care” describes how the IHC providers position the role of IHC collaboration within conventional care and in a wider health care discourse. The IHC team wants to both collaborate and be part of conventional medicine but they also want to bring something new to the field and be able to be part of changing and improving health care. This is an evolving process: during one of the first patient conferences, one of the providers begins to discuss the importance of defining a shared vision of IHC:

Excerpt 8

CAM provider 1—It is our goal, but I do not know that we have a creative goal, a little bit more, that involves a more demanding goal. And that is something to bring up in the discussion? Because we are for once arguing the case whether this is a worthwhile thing, right? So, I think that it is important that we make an explicit and brave description of what we stand for.

Excerpt 8 shows how CAM provider 1 is bringing up a discourse on the importance of a shared vision of IHC. The CAM provider is not clear about what the vision should be, but suggests that a brave and demanding vision of what the IHC team stand for is important. By saying that they have to argue the case, the CAM provider is referring to their current position within the conventional system and a wider medical discourse.

In the middle of the patient conferences, the physician begins to discuss the role of IHC in future in pain rehabilitation within conventional care by suggesting that more people involved in conventional care would benefit from collaborating with CAM providers. When needed, the physician in the IHC team referred patients to relevant professionals within conventional care. Even so, the IHC team notices that there might be a need for a broader and more established IHC team/network than the present project to fulfill all patients´ needs, for example with psychologists and dietitians. At different times during the patient conferences, the IHC team encounters collaborative shortcomings in relation to conventional health care.

Excerpt 9

CAM provider 1: What she said, she has a physiotherapist, some treatment, so she told them (the physiotherapist) that she goes to a CAM provider. I believe it is a good thing to bring this up. It is a typical situation, I can say. But, ahh, if you go there, then you have to stop here with us. For her it was no big deal, because she thought that the CAM therapy was good, but she thought it was a shame it had to be an either or situation, so to say. So she asks why?

In excerpt 9, CAM provider 1 describes a situation of collaborative shortcomings. When CAM provider 1 says that it is a typical situation, it is seen in the discourse that this is a situation the CAM provider has encountered before. He continues to emphasize that the patient is satisfied with the CAM therapy and that she does not want to have to choose (between conventional and complementary health care).

During the last patient conferences, the IHC team develops the discourse to include how they position the role of IHC within the wider health care discourse.

Excerpt 10

CAM provider 1- *It is almost our duty to come up with new suggestion*, *a new model for measurement*. *Otherwise*, *we can easily be thrown out*, *nobody has tried it*, *so we have to find new rules for measurement*. *To a whole new*, *otherwise… we will not get any space*.

In excerpt 10, CAM provider 1 describes a practice on the margin of conventional health care and suggests that they (IHC) might be thrown out or that they might not get any space. The providers continue to discuss factors that would facilitate collaboration:

Excerpt 11

CAM provider 1- Education is an important aspect, and maybe, we can work towards a new policy for collaboration on the work place.

CAM provider 6- it is a paradigm shift and we need to measure health with new instruments than for example pain relief, which is an incredibly delimited way to measure health.

In excerpt 11, the discourse includes different aspects to strengthening the role of IHC, for example education, policy change and how to best document and evaluate the effect of IHC. The IHC team also suggests that patients on a long sick leave (calling them people in the gray zone) are the ones that can best be helped by integrative care. By the time of the final patient conference, the IHC team has developed the discourse about their vision of IHC provision:

Excerpt 12

CAM provider 1- *I hope we can develop in a larger context…*, *that we can give it other priorities*, *another way of seeing*, *another way of viewing the whole*. *What is sickness*? *What is healing*? *What is the healing process*? *… that we can bring something new to the field*.

In excerpt 12, the CAM provider expresses some questions about a wider view of health and healing (than in conventional care), summarizing the discourse about a vision of integrative care. The IHC team is convinced that they have something new to offer the health care system.

## Discussion

### Discussion of results

In the present study, the IC team moved their subject positions from individuals treating subjects to subject positions as team members, as they gradually formed a group identity. Initially, the CAM providers positioned themselves as individuals treatment subjects to define their individual roles in the study and the physician took a leading role in the decision-making process. The role of the physician might have been a consequence of the design of the project, were the patients were referred to the IHC team from the conventional care system. In a case study of a panel discussion, Anderson [16] has shown how different mainstream and alternative practitioners limited themselves to a language of bio-medicine instead of using their different theories of health. In contrast to the biomedical dominance presented by Anderson [16], the IHC team in the present study managed to develop a non-hierarchical, consensus-based decision-making process. The longitudinal design of the study where the providers were able to learn about each other’s methods facilitated the process. Aspects such as describing and recognizing one´s own role and at the same time respecting and recognizing other professions’ responsibilities and competences are described as important collaborative competencies [34].

During the discourse, the IHC team began to position themselves as members of a team. They capitalized on the synergies in their collaboration as they came to recognize how they learned from each other and they also explained how they perceived how patients benefit from the collaboration. Gabory [35] suggests that providers’ belief in the benefits of collaboration correlate with the extent of the providers’ trust, cooperation and exchange of knowledge. According to previous research [18], the fact that providers were experienced and that some of them had training in several therapies, and also that they held properly organized meetings with all providers seems to have facilitated collaboration. In spite of this, the IHC team needed quite some time to adapt to the unusual mode of interdisciplinary dialogue and collaboration. This might be due to the complex nature of IHC collaboration with providers from different health paradigms. The Academic Consortium for Complementary and Alternative Care in America has developed a competency document with the aim of fostering collaboration between CAM and conventional health care professions and they have recognized the need to strengthen professional identities among CAM professions through for example inter-professional education [36].

The IHC team in the present study identified collaborative shortcomings in relation to conventional care. Hollenberg [37] suggests that macro- structural determinants have much greater effect on the practice of integrative medicine than on inter-professional collaboration within conventional health care, because CAM is situated mainly outside of the conventional health care system. Different countries have different ways to integrate CAM and conventional health care [12]. In the European Union, Wiesener et all [38] show a diversity in the legal and regulatory status of complementary and alternative medicine among the countries, where Sweden has no general legislation governing complementary and alternative medicine (CAM). Most CAM therapies, also those included in this study, are typically practiced outside of the conventional health care system in Sweden. One exception is naprapathy, which is a licensed health care profession in Sweden [39]. This was shown in the discourse, as naprapathic manual therapy was perceived as a profession linking the complementary and conventional health care systems. Soklaridis et al. [40] suggest that the exclusion of CAM from public funds does not support the development and integration of IHC and Sundberg et al [4] argue that a maintained polarization between complementary and conventional health care may be unhelpful for the patients and might even be dangerous from a patient safety perspective. Gaboury et al.[41] discuss the terminology of CAM and IHC and points out that the term CAM needs to be acknowledged as part of the whole in the field of research and practice. Furthermore, they [41] discuss the transition of CAM into IHC, where the term IHC emphasizes a diversity of therapeutic options without any differentiation between different health paradigms.

The discourse revealed an antagonistic relationship toward the conventional health care system, where the IHC team described how they want to collaborate but are excluded from the conventional health care system. Similar results have been found among traditional medicine providers (TCM) [42]. The TCM providers were open to collaboration with western medicine, but they were also afraid that western medicine would overshadow the TCM and therefore concerned about how to best integrate TCM and western medicine [42]. The results show how the IHC team negotiates the role of IHC within conventional care and in a wider health care discourse. The antagonistic relationship can be understood as part of forming a group, where the “othering” is used to stabilize and understand the boundaries of the group [26]. The IHC team discussed the importance of a definition of IHC and drew up a common goal for IHC. This construction of a common goal, together with the formation of a collective identity, is what Belenger and Rodriguez [43] point out as a fundamental aspect of team formation. Previous research [44] shows that providers in Australia describe a lack of a clear definition of integrative medicine, but that a common philosophy and trust in the involved providers was important for IHC practice. The IHC team in this study wishes to change the hegemony in the health care system to include their vision of a more holistic and patient-centered care. To do so, they wish for a broader and established IHC collaboration to fulfill patients´ needs. In society, due to growing levels of chronic illnesses with escalating health care costs, there is a growing demand for high quality and safe health care services that are individualized and person-centered. IHC is a response to the increasing use of CAM, growing out of a concern that the concurrent use of conventional health care and CAM without adequate knowledge on the part of providers might jeopardize patient safety and satisfaction. The IHC team in this study formed a vision where they aimed to meet this growing demand for person-centered health care. The WHO recognizes that some of the important factors in shaping effective collaborative practices within health care are to change the culture and attitudes of health workers, to have a willingness to update, renew, and revise existing curricula and have appropriate legislation that eliminates barriers to collaborative practice [1].

### Discussion of methods

Discourse-theoretical studies commonly work with contextualized material, exploring the construction of a particular problem in a specific context [33]. The material in this study did not include such contextualized material. The data consisted of reactive, linguistic data [26], a specific micro-level setting of interaction to explore the aim of the study. West [45] argues for paying closer attention to the materiality and interactions in local practice situations as this type of material can provide for a thick description of practices in discourse studies.

There were a number of difficulties in transcribing the material, e.g. hearing exactly what each provider was saying, especially when several people were speaking at the same time. Most of these issues were resolved by repeated listening. The transcription level was on making as exact transcriptions as possible of the spoken language. In some discourse analysis, which focuses more on the linguistic analysis, a more detailed transcription method is necessary. It is possible that such transcript and analysis would have revealed more details in the interactions, but this was not the objective of the study.

In the process of data analysis, the themes were initially identified by the first author and subsequently discussed with the other authors to arrive at themes that clearly described how collaboration within the IHC group developed over time. The first author had participated in the last of the patient conferences as an observer and was able to use field experience in the analysis. The second author had participated in all patient conferences and was able to discuss the themes in depth.

Although the legal status of IHC and the regulation of CAM differ between different countries and may thus not be transferrable across borders [38], the results of the current study might be transferable to other contexts on a conceptual level, regarding for example ways of how providers engage in collaborative learning and interchange knowledge and ideas over time in clinical shared practice.

## Conclusion

The IHC process of collaboratively managing patients with back and neck pain involved the gradual formation of an IHC team identity, which increasingly facilitated interdisciplinary, non-hierarchical consensus-based decision-making in the formulation of treatment plans. From the IHC providers’ perspective, this seems to have advantages for both the IHC providers and the patients. However, the current discourse might imply that a change in the legal and organizational aspects of the health care system would be needed for IHC to be sustainable in Sweden.

### Further research

This study focused on IHC interaction and collaboration, an analysis of what treatment strategies the IHC providers in pain rehabilitation agree would deepen the understanding of how to best treat patients with back and neck pain. The discourse also revealed the importance of contextual factors in IHC collaboration. This aspect could be further investigated by analyzing relevant legal, social, historical, and political factors and their impact on IHC collaboration and provision.
